# Neonatal outcomes of Syrian refugees delivered in a tertiary hospital in Ankara, Turkey

**DOI:** 10.1186/s13031-015-0066-1

**Published:** 2015-12-21

**Authors:** Mehmet Büyüktiryaki, Fuat Emre Canpolat, Evrim Alyamaç Dizdar, Nilüfer Okur, Gülsüm Kadıoğlu Şimşek

**Affiliations:** Neonatal Intensive Care Unit, Zekai Tahir Burak Maternity Teaching and Education Hospital, Hamamönü, 06230 Ankara Turkey

**Keywords:** Refugees, Syrian, Neonate, Economic burden

## Abstract

We retrospectively reviewed the medical records of all Syrian immigrants from the TurkishSyrian border who delivered the Zekai Tahir Burak Maternity and Teaching Hospital Neonatal Intensive Care Unit (NICU) in Ankara, Turkey. Between January 2013 and December 2014 a total of 36,346 women gave birth at this center. Of these, 457 women were Syrian immigrants, comprising 1.2 % (457/36,346) of all deliveries. The number of births among Syrian refugees in Turkey appears to be increasing. Further research is needed to understand the relative morbidity of babies born to Syrian refugees compared to the local population, as well as the economic impact on facilities treating these cases.

## Correspondence/findings

Two years have passed since the conflict in Syria began; it has cost thousands of lives, and injured and displaced millions. This conflict has caused millions of Syrians to flee, seeking safety in Turkey. It is estimated that 3 million immigrants from Syria have come to reside in Turkey since March 2011 [1, 2]. According to reports from the Disaster and Emergency Management Agency of the Turkish Government, by July 2014, 1,000,103 Syrian refugees were registered in Turkey [1, 2]. These immigrants reach Turkey by escaping through the Syrian-Turkish border, and in the last 6 months their numbers have nearly doubled. The Turkish Government prepared refugee camps and villages near the Syrian border but most of the immigrants moved from the border to reach larger cities, namely Ankara, the capital city, Istanbul and Izmir. These immigrants lack medical insurance and financial support and, therefore, have reduced access to pre- and post-natal care. Moreover, the medical treatment of this population is further complicated as most of them do not speak Turkish or English.

In the literature, there are well-established disparities in prenatal outcomes of minorities living among different native populations; these include higher rates of low birth weight, preterm deliveries, perinatal mortality and congenital anomalies [3–7]. Reports from different centers have shown that refugees are susceptible to adverse perinatal outcomes [8]. Furthermore, female refugees of child bearing ages are more likely to be victims of traumatic events, which may lead to an increased risk of preterm birth [9].

Zekai Tahir Burak Maternity and Teaching Hospital NICU is a level 3 neonatal intensive care unit which provides health care for especially high risk babies all around the Turkey. It is located in the capital city, Ankara. We retrospectively reviewed the medical records of all Syrian immigrants from Turkish-Syrian border who delivered in this center. Between January 2013 and December 2014 a total of 36,346 women gave birth at the center. Of these, 457 women were Syrian immigrants, comprising 1.2 % (457/36,346) of all deliveries. The number of Syrian women delivered by caesarean section was 162 (36 %). The mean age of the mother was 26 (minimum 14, maximum 44 years). Of those women giving birth who were 41 years of age and below, the mean age was 18 years. Four hundred and fifty seven infants were included in the data analysis; 74 % were term, 26 % were preterm. Of these infants, 10 infants were low birth weight infants, and 4 were extremely low birth weight infants. Fifty infants were admitted to the intensive care unit. The most common symptom for NICU admission was respiratory distress. The overall mortality rate of the infants was 1.8 %. The Turkish neonatal mortality rate is 0.04 % according to World Health Organization Reports and the Ministry of Health of Turkey (http://www.saglik.gov.tr/TR/dosya/1-97020/h/saglik-istatistik-yilligi-2013.pdf). The total cost for these deliveries and NICU admissions was $322,857 USD over 2 years. Health care provided to refugees in Turkey is either covered by the government or by the hospitals themselves (which discharge refugee patients without receiving payment).

The number of Syrian immigrants delivered at our center has reached a substantial level and can no longer be overlooked or disregarded. In the year following collection of these data, the numbers more than doubled, and today the Syrian population accounts for more than 2 % of all deliveries in Turkey.

In summary, there is an increasing number of birth of Syrian refugees in Turkey. In our view, morbidity among Syrian refugee infants appears to be higher than that of local infants but further data are required to know whether this is indeed the case. Treating these infants presents an economic challenge for Turkish health care facilities that must be addressed. Our aim is to highlight this issue and urgently call on the international community to co-operate toward improving financial and political support for the Syrian refugee crisis.
