# Erratum to: Neonatal outcomes of Syrian refugees delivered in a tertiary hospital in Ankara, Turkey

**DOI:** 10.1186/s13031-016-0091-8

**Published:** 2016-07-29

**Authors:** Mehmet Büyüktiryaki, Fuat Emre Canpolat, Evrim Alyamaç Dizdar, Nilüfer Okur, Gülsüm Kadıoğlu Şimşek

**Affiliations:** Neonatal Intensive Care Unit, Zekai Tahir Burak Maternity Teaching and Education Hospital, Hamamönü, Ankara 06230 Turkey

## Erratum

Unfortunately, the original version of this article [1] contained an error.

The sentence “The Turkish neonatal mortality rate is 0.04 % according to World Health Organization Reports and the Ministry of Health of Turkey (http://www.saglik.gov.tr/TR/dosya/1-97020/h/saglik-istatistik-yilligi-2013.pdf)” should have read “The Turkish neonatal mortality rate is 0.4 % according to the Ministry of Health of Turkey (http://www.saglik.gov.tr/TR/dosya/1-97020/h/saglik-istatistik-yilligi-2013.pdf)”.

The corrected sentence has been included in full in this erratum.
